# Neuromuscular Electrical Stimulation to Combat Muscle Atrophy During Spaceflight: A Narrative Review of Mechanisms and Potential Applications

**DOI:** 10.3390/life16020258

**Published:** 2026-02-03

**Authors:** Broderick L. Dickerson, Ryan J. Sowinski, Drew E. Gonzalez

**Affiliations:** 1 Exercise and Sport Nutrition Lab, Department of Kinesiology and Sport Management, Texas A&M University, College Station, TX 77843, USA; rjs370@tamu.edu (R.J.S.); or drewgonzalez418@shsu.edu (D.E.G.); 2 Department of Kinesiology, School of Natural Sciences, St. Edwards University, Austin, TX 78704, USA; 3 Occupational, Performance, and Nutrition Laboratory, Department of Kinesiology, Sam Houston State University, Huntsville, TX 77340, USA

**Keywords:** microgravity, astronaut, muscle, anabolism, atrophy, stimulation

## Abstract

As humanity continues to strive for extraplanetary exploration, which is quickly gaining marked governmental and industrial support and recognition, there are still substantial detriments to astronaut health during long-duration spaceflight (i.e., muscle atrophy) that must be addressed. The effects of long-duration spaceflight on muscle architecture, morphology, and function have been well documented since the Apollo and Space Shuttle Programs. Countermeasures focused on resistance or aerobic training, such as the Advanced Resistive Exercise Device, Multi-modal Exercise Device, flywheel exercise, and aerobic exercise on a mounted treadmill and/or a cycle ergometer with vibration isolation system, have been assessed to combat the functional and mechanical losses in muscle while astronauts are in low Earth orbit. However, a lesser-understood countermeasure to muscle atrophy during spaceflight is neuromuscular electrical muscle stimulation (NMES). Although utilization in spaceflight is limited, ground-based research on NMES in diseased or injured populations demonstrates its effectiveness as a promoter of muscle anabolism and growth. The previous literature has suggested the use of electrical muscle stimulation as a low-effort modality of exercise for astronauts, which could effectively enhance astronaut health and contribute to mission success. The efficacy and mechanisms of action of using NMES to attenuate atrophy in astronauts will be discussed in this review.

## 1. Introduction

As humanity ventures back to the moon and further onto Mars, extravehicular activities (EVAs) will depend upon an astronaut’s ability to produce adequate muscular force and move freely in a microgravitational environment to complete various operational tasks. It is well established that an exposure to microgravity or a ground-based analog for extended durations induces muscle atrophy (wasting) with concomitant losses in force production [1,2,3], which can lead to overall deficits in muscle functionality upon re-entry to earth orbit. One of the more heavily studied ground-based analogs for long-duration spaceflight (LDS) is long-duration bed rest, wherein an individual lies supine for an extended period (e.g., up to or over 30 days) [4,5,6,7]. Another related analog, pioneered by Russian bioastronautics investigators [8], is the head-down tilt (HDT) procedure [9]. HDT has been implemented across various study durations to simulate the effects of microgravity on the musculoskeletal and cardiovascular systems by unloading the resistive effects of gravity [10,11]. HDT causes a cephalic fluid shift and offloads weight bearing, impairing blood flow to the lower extremities, resulting in a loss of muscle and bone mass, similar to what is seen in astronauts after LDS [10,11]. In addition to spaceflight itself, dry immersion, cast immobilization, unilateral lower limb suspension, and rodent hindlimb unloading have been employed as other analogs of interest to investigate muscle wasting and pragmatic countermeasures [12,13,14,15,16]. Nonetheless, it appears that muscle architecture and the contractile apparatus are either altered or compromised to some degree, regardless of the methodology used to induce microgravity-like conditions. Our understanding of the compromised muscle, as well as the rate of degradation or impairment, has helped shape the potential countermeasures that may be taken to offset or at least maintain as much skeletal muscle mass as possible under these extreme microgravitational conditions.

Space exploration programs have placed a great importance on the development of interventions to mitigate muscle loss in microgravity during LDS. For future long-duration missions, the National Aeronautics and Space Administration (NASA) plans to transition its focus from low Earth orbit (LEO) to lunar and Mars-based missions. Missions beyond LEO will include mass and volume constraints on the exercise modalities that can be used on the shuttle transporting astronauts, thus establishing a need for an implementable countermeasure that adheres to these constraints. Nevertheless, over the years, several countermeasures have been incorporated into spaceflights to attenuate muscle atrophy, maintain skeletal muscle mass, inhibit bone resorption, and mitigate cardiovascular deconditioning. For instance, the Advanced Resistance Exercise Device (ARED), consisting of vacuum cylinders and flywheel-like assemblies, was developed to enable astronauts to perform high-intensity resistance exercises with variable resistance loading and a full range of motion [1,17], and is currently aboard the International Space Station (ISS). Additionally, flywheel systems can be used for resistance or endurance exercise via imposing high-load concentric or eccentric muscle forces [1,17]. Specifically, the multimodal exercise device (M-MED), a flywheel exercise machine, provides endurance and resistance-based exercises [18,19]. These types of exercise systems have been shown to mitigate musculoskeletal and cardiovascular deconditioning in ground-based analogs [18,20]. Generally, flywheel exercise systems offer low-volume designs, an advantage these apparatuses have over other traditional exercise devices historically implemented on the ISS due to their limited space occupancy. Additionally, human-powered short-arm centrifugation can be used to impose a hypergravitational stimulus on the astronaut; however, its feasibility of incorporation into spaceflight is debated [21]. Lastly, blood flow restriction training (BFRT) has also been integrated into microgravity simulations and is currently being studied on the ISS, even being shown to enhance muscular responses to exercise during parabolic flight [22]. The design of BFRT equipment also offers astronauts and researchers a low-volume configuration in its utilization, therefore strengthening the efficacy of its implementation as a plausible exercise countermeasure in future missions. Spaceflight applications of BFRT as an exercise countermeasure have been explored recently [23,24].

A well-supported potential countermeasure in ground-based studies that has been scarcely utilized in spaceflight is neuromuscular electrical muscle stimulation (NMES), which involves placing electrodes in or on skeletal muscle to pass electrical stimuli to motor neurons that innervate muscle fibers, causing pulsatile muscle contractions [25,26,27]. This can help preserve muscle mass and strength, or even elicit muscle growth in certain types of muscle fibers [28]. These effects have been demonstrated in studies from various research foci, including aging [29,30,31], paraplegia [32,33,34,35,36,37,38,39,40], disease [39,41,42,43], and animal meat science [39,41,42,43]. Electrical stimulation has been utilized in clinical practice as a retraining, rehabilitative, and/or ergogenic tool for individuals undergoing post-operative rehabilitation [44,45,46] or athletes recovering from injury [47,48,49,50]. Additionally, NMES has been shown to combat sarcopenia-induced muscle atrophy and has shown promise in long-term, bedridden elderly individuals [51,52,53,54,55]. A major benefit of using NMES onboard either the ISS, future space stations, or on a shuttle transporting astronauts to the moon or beyond is the limited mass and volume taken up by the NMES equipment. Recently, NASA has outlined that there will be configuration constraints for long-duration exploration vehicles including size, mass, and power capacity for an exercise system, thus emphasizing the need for alternative exercise countermeasures. The ARED and flywheel exercise device (i.e., M-MED) systems are hallmark exercise apparatuses aboard the ISS; however, they occupy the potential space and power needed for other resources that will be emphasized in future missions [56,57,58]. Therefore, if one or more of these devices are omitted in future spaceflights, NMES can be utilized as an adjunct countermeasure.

## 2. Methods

A narrative review with a systematic approach was used to collect eligible studies for this manuscript. A comprehensive search of four databases—PubMed, Google Scholar, ScienceDirect, and publicly available NASA research archives—was conducted between January 2025 and November 2025 to ensure the inclusion of possible recent findings. Researchgate was used for full-text retrieval.

Studies were included if they examined the effects of electrical stimulation on skeletal muscle preservation or adaptation. Eligible studies utilized any form of electrical stimulation, NMES, functional electrical stimulation (FES), transcutaneous, percutaneous, implanted, or garment-integrated systems. Studies employing both low- and high-frequency stimulation protocols were considered. Additionally, included studies spanned relevant experimental and applied contexts, including microgravity exposure, ground-based analogs of spaceflight (e.g., bed rest, unloading, dry immersion), disease or disuse conditions, and musculoskeletal injury or rehabilitation settings. Studies were eligible whether electrical stimulation was applied as a standalone modality or in combination with nutritional support or concurrent exercise training, provided muscle mass, muscle morphology, or functional activity outcomes were reported. In addition to human studies, animal and in vitro cell culture models were included if they provided mechanistic insight into the molecular and cellular pathways through which electrical stimulation influences skeletal muscle adaptation. Exclusion criteria included studies that had a non-English origin; did not report skeletal muscle outcomes relevant to preservation, morphology, or function; used NMES solely for diagnostic or neuromodulatory purposes without the intent to elicit skeletal muscle adaptations; focused exclusively on cardiac or smooth muscle tissue; and consisted of editorials, abstracts, master’s theses, and commentaries that did not provide original data.

The following search terms and Boolean operators were employed in varying combinations across databases: “anabolism”, “application”, “astronaut health”, “bed rest”, “calpains”, “cathepsins”, “dry immersion”, “electrical stimulation”, “functional electrical stimulation”, “head-down tilt”, “hypertrophy”, “injury”, “long-duration spaceflight”, “microgravity”, “microRNA”, “mTOR”, “muscle atrophy”, “muscle protein breakdown”, “muscle protein degradation”, “muscle protein synthesis”, “myogenic regulatory factors”, “neuromuscular electrical stimulation”, “sarcomere”, “sarcopenia”, “satellite cells”, “signal transduction”, and “water immersion”.

## 3. Spaceflight and Muscle Degradation

**Figure 1:**
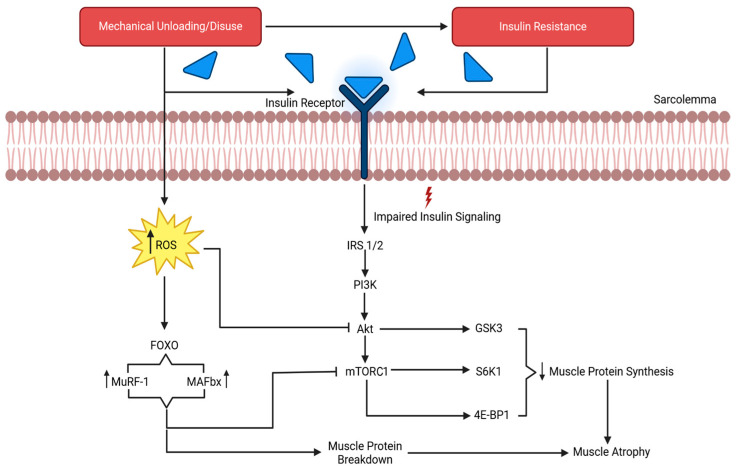
The results of mechanical unloading on the molecular process of muscle degradation. IRS 1/2, insulin receptor substrates 1 and 2; PI3K, phosphatidylinositol 3 kinase; Akt, protein kinase B; mTORC1, mechanistic target of rapamycin complex 1; GSK-3β, glycogen synthase kinase 3β, S6K1, ribosomal protein S6 kinase 1; 4E-BP1, 4 eukaryotic initiation factor binding protein 1; ROS, reactive oxygen species; MuRF1, muscle RING-finger protein 1; MAFbx/atrogin-1, muscle atrophy F-box. Adapted from Gao et al. [64].

Data from the Skylab missions suggest that spaceflight can induce a loss of up to 20% in muscle strength within a 1- to 2-month mission duration [59,60], which begins with an imbalance in muscular protein turnover [61,62]. Muscle protein stability depends on the relationship between synthesis and degradation. However, during spaceflight, the absence of mechanical loading, combined with an insufficient caloric and protein intake, reduces muscle protein synthesis (MPS) and increases muscle protein degradation (MPD), resulting in muscle atrophy. MPD is a complex and energy-dependent process involving various hormones, lysosomes, and hydrolytic enzymes. Figure 1 provides a general overview of the mechanisms underlying atrophy due to muscle (mechanical) disuse or unloading, with signaling molecules targeting downstream pathways that inhibit MPS. Moreover, insulin and insulin-like growth factor 1 (IGF-1) are integral upstream mediators of MPS and MPD via the inhibition of the ubiquitin–proteasome pathway [63]. In fact, Gao and colleagues [64] reported that insulin resistance promotes MPD by impairing insulin signaling through the reduced activity of insulin receptor substrates 1 and 2 (IRS-1 and IRS-2). Short- and LDS have been associated with an increased risk of insulin resistance in astronauts [65,66], suggesting this as a potential mechanism of MPD that can lead to muscle atrophy. Furthermore, one’s diet is another important consideration, wherein caloric intake and adequate amino acid consumption are primary drivers of MPS. As observed in Apollo and Space Shuttle crew members, insufficient caloric and protein intake led to caloric deficits and a negative nitrogen balance, thereby promoting muscle protein degradation and a loss of body mass [67,68,69]. However, more recently, astronauts have been able to mitigate losses by maintaining an adequate caloric and amino acid intake, allowing them to sustain, or even increase, energy expenditure due to the appropriate incorporation of space food systems and exercise countermeasures [67,68,69]. Therefore, optimal dietary intake during spaceflight should not be overlooked when considering all potential factors that may contribute to muscle atrophy from LDS.

Prolonged mechanical unloading has been linked to impairments in key protein synthesis-related pathways, including the mechanistic target of rapamycin complex (mTORC) and mitogen-activated protein kinase (MAPK) signaling. For example, IGF-1 signaling is impaired with prolonged unloading, which promotes insulin resistance and disrupts mTORC signaling [70]. This signaling pathway is a master kinase regulator for peptide chain initiation, subsequent mRNA translation, protein synthesis, and ultimately anabolism [64]. The inhibition of mTORC suppresses the phosphorylation of its downstream targets, directly reducing mRNA translation and blunting protein synthesis, as evidenced by in vitro simulated microgravity studies [71,72]. Furthermore, the suppression of extracellular signal-regulated protein kinase 1 and 2 (ERK1/2) exacerbates this response in muscle. ERK1/2 is a part of the MAPK family and is responsible for conveying extracellular signals to the nucleus to regulate the cell cycle, ultimately mediating adaptations to stimuli [73]. Studies show that ERK1/2 are sensitive to simulated microgravity and mediate molecular responses in myocytes [74,75]. Spaceflight suppresses ERK1/2 activity, reducing the MAPK-mediated transcriptional activation of anabolic genes. This blunting of transcriptional signaling contributes to net protein loss, particularly in type I muscle fibers [1]. Type I muscle fibers may experience a greater net protein loss during microgravity due to several interrelated mechanisms. Slow-twitch fibers are chronically active under normal gravitational conditions and therefore undergo a proportionally larger reduction in mechanical loading and tonic activation during unloading [60,76,77]. Secondly, the maintenance of the type I phenotype relies heavily on ERK1/2–MAPK-dependent transcriptional signaling, which is suppressed under microgravity conditions [78], disproportionately reducing anabolic gene expression in oxidative fibers, possibly due to the shift from type I muscle fiber types to type II. Additionally, type I fibers exhibit higher basal protein turnover rates; thus, the unloading-induced suppression of protein synthesis more rapidly shifts the net balance toward degradation [79,80]. Finally, type I fibers are particularly sensitive to unloading-induced insulin resistance and oxidative stress, which attenuate Akt–mTOR signaling and promote the FoxO-mediated activation of proteolytic pathways. Collectively, these mechanisms contribute to preferential net protein loss in type I fibers and a shift in phenotype from type I to type II during spaceflight and its ground-based analogs.

As these gene expression-related pathways for protein synthesis become affected by the catabolic state induced by a microgravity environment, calpains will begin to initiate MPD. Calpains are Ca^2+^-dependent cytosolic proteases that degrade structural proteins into fragments, priming them for ubiquitination, a process heightened during muscle degradation [81,82]. Specifically, calpains degrade desmin and titin, integral filament proteins that regulate sarcomere architecture and muscle integrity, thereby deconstructing the sarcomere structure [81]. Additionally, cathepsins, which are similar in function to calpains, are lysosomal proteases that degrade actin and myosin proteins, thereby eliciting the loss of myofibrillar proteins, primarily in muscle unloading conditions [83]. Cathepsins are categorized into various subtypes and differ based on the site of protein degradation and tissue expression [84]. In particular, cathepsins S, E, F, B, and K have been implicated in various forms of skeletal muscle atrophy [84,85,86,87,88,89]. Cathepsin S appears to be one type that contributes to muscle repair and regeneration [90,91]. Animal models have assessed chronic stress-induced skeletal muscle atrophy and dysfunction, noting that a deficiency or inhibition in cathepsin S is associated with reduced muscle damage, metabolic imbalance, and apoptosis [92,93]. Hou et al. speculated that cathepsin S likely acts as a context-dependent muscle modulator, aiding regeneration under repair conditions, but exacerbating muscle atrophy under pathological conditions, while also noting that cathepsin E likely plays a critical role in the influence of the immune system on muscle health, whereas cathepsins F and B appear to promote muscle degeneration [89,94,95,96]. Particularly, cathepsin F may target lysosomes through the signaling of peptide-independent pathways [97], where cathepsin B has been linked to proteolytic pathways that lead to muscle protein degradation [98]. In addition, cathepsins cause autophagy–lysosomal degradation coupled with proteasomal protein breakdown, which is initiated by calpains [84]. Calpains and cathepsins work in tandem with ubiquitin ligases, offering an integrated system for muscle protein degradation. Evidence from spaceflight and analogous research models in rodents suggests modifications in calpain and cathepsin activity, although the results appear to be unclear. Ikemoto and scientists [99] observed significantly increased cathepsin mRNA concentrations after 16 days of spaceflight and 10 days of hindlimb unloading, with no significant change in calpains, thereby suggesting that muscle protein degradation is increased. Another study, albeit in cardiomyocytes, showed no changes in the markers of MPD in simulated microgravity [100]. Meanwhile, Belova et al. [101] showed enhanced calpain mRNA levels in soleus tissue after hindlimb unloading in rats. Other studies also show increased calpain activity, concomitant with the degradation of desmin, in skeletal muscle during the first days of hindlimb unloading [94,95,96]. However, these studies did not utilize physiological concentrations of Ca^2+^ in their experimental media, which could have affected the results.

Calpains and cathepsins facilitate MPD, along with ubiquitin ligases, which are protease-like ligands that mark proteins for proteasomal degradation by covalently attaching themselves to the proteins, signaling to proteasomes for hydrolytic degradation [102]. The mRNA expression of ligases such as muscle RING-finger protein 1 (MuRF1) and muscle atrophy F-box (MAFbx/atrogin-1) is increased with atrophy-inducing conditions [103,104]. Corroborating this, Shimkus and researchers [105] exposed rats to 28-day hindlimb suspension and found increased MuRF-1 and MAFbx/atrogin-1 expressions in hindlimb skeletal muscle. Muscle sizes were also smaller in the suspended rats. Baehr and workers [106,107] also assessed MuRF1 expression in rats subjected to hindlimb bilateral control or MuRF1 transfection prior to denervation, wherein MuRF1 transfection resulted in a higher MuRF1 expression and induced a loss in muscle mass after 14 days. Beyond these proteolytic pathways, unloading and microgravity also alter muscle phenotype and neuromuscular characteristics, resulting in well-documented fiber-type transitions [108].

Generally, type I muscle fibers respond the most dramatically to microgravity, with type I myosin heavy chains shifting to type II [72]. Numerous alterations to neuromuscular structure and function also accompany this shift in muscle fiber type [109,110]. Concurrent with this are alterations in myosin heavy chain (MHC) isoforms after microgravity. Type I MHC isoforms are significantly reduced after spaceflight [111,112]. However, it is mainly unknown if type I motor neurons decrease entirely or exhibit a strong shift towards type II. In addition, research from Trappe et al. [109] does show a decrease in type I MHC expression but also an increase in MHC I/IIa hybridized fibers in the gastrocnemius and soleus muscles of astronauts after six months of spaceflight. Reductions in Type I/IIa expression from the soleus muscle were mirrored in other research [113]. The alterations of the hybrid muscle fibers might be explained by the effects on the type I, slow-twitch isoforms of MHC. One mechanism that may help explain this adaptation is the loss of ERK1/2 activity function in microgravity and analogous conditions due to a loss in a muscle’s ability to regulate transcription and eventual protein synthesis. ERKs, a type of MAPK, mediate gene expression via mechanotransduction from the sarcolemma to the nucleus, and a loss in this ability might impair cell growth and eventual protein synthesis. Lastly, a reduction in MAPK expression can blunt transcription specific to the antigravity muscle fibers [1,3]. Overall, spaceflight-induced muscle atrophy results from a combination of decreased anabolic activity, the activation of various protein breakdown systems, and structural changes in muscle fibers. Grasping these interconnected mechanisms is crucial for developing strategies to maintain strength and metabolic health during extended missions.

## 4. Utilizing NMES in Disease and Microgravity

**Figure 2:**
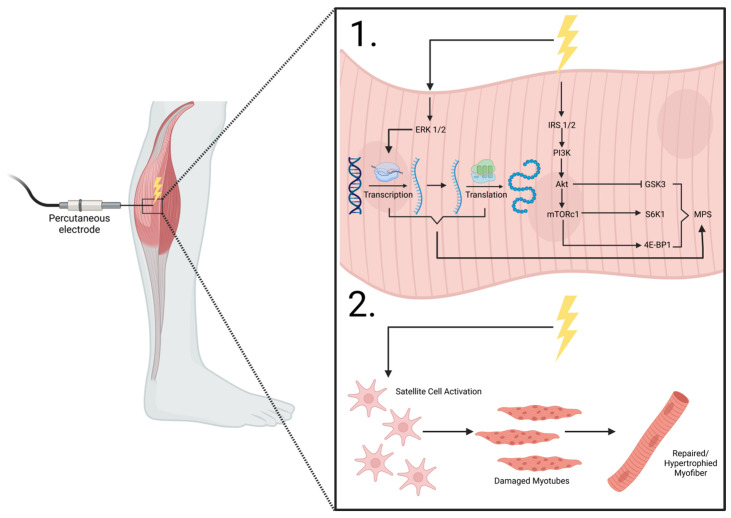
NMES percutaneous electrode placement. 1. shows the intracellular effects of electrical stimulation on the anabolic machinery and mTORC1 signaling and transcription/translation modifications that lead to MPS. 2. shows the purported effects of electrical stimulation on myocyte satellite cell activation, enhancing reparative mechanisms on damaged myofibers, leading to repaired/hypertrophied muscle cells. ERK1/2, extracellular receptor kinase 1/2; IRS 1/2, insulin receptor substrate 1/2; PI3K, phosphoinositide 3-kinase; Akt, protein kinase B; mTORC1, mechanistic target of rapamycin complex 1; GSK3, glycogen synthase kinase-3; S6K1, ribosomal protein s6 kinase B1; 4E-BP1, eukaryotic initiation factor 4E-binding protein 1; MPS, muscle protein synthesis. Adapted from MacDiarmid et al. [117].

NMES involves applying low- to high-frequency (20–50 Hz) currents to induce muscle tetany and contraction and can be used in instances of disuse or disability [114]. Primarily, NMES use falls into two categories: transcutaneous or implanted [115]. Transcutaneous NMES involves electrode placement on the skin’s surface, whereas implanted NMES involves intraneural, epineural, percutaneous, or epimysial electrode placement. Some cited studies—including those involving complete denervation—utilize FES and long-term implanted stimulation, which aim to primarily improve functionality in movement and are utilized for neurological rehabilitation [116], though results from studies using NMES vs. FES modalities are similar. Figure 2 depicts the reported mechanisms associated with percutaneous electrode placement on the lower leg musculature.

It is plausible that NMES can help preserve certain muscle phenotypes and delay the conversion of type I to type II fibers [115], as astronauts will be engaging in other forms of exercise while maintaining an adequate caloric and protein intake. Table 1 shows the results from studies using various forms of NMES (transcutaneous, percutaneous, intramuscular, etc.) as a countermeasure for muscle wasting, which has been offered as a rehabilitative and training technique for various populations [34,35,36,45,49,50,53,54,114,115,118,119,120,121,122]. Moreover, Boncompagni and researchers [123] conducted a long-term (2.4–9.3 years) FES study in patients with spinal cord injuries who had experienced a complete denervation of their lower limbs. Electrical stimulation almost completely restored the muscles’ ultrastructural architecture (sarcomere organization, triad integrity, myofibrillar alignment) and muscle mass in the lower extremities, even in patients who had experienced long-term denervation (>2 years), further highlighting the potential benefits of the electrical stimulation method. These results were mirrored in other studies by the same research team in the same population of patients [124,125]. In one study, a long-term (2 years) home-based FES program (5 d/wk, biphasic 120–150 ms/stimulation, 80 V) was implemented on the quadriceps of participants who suffered permanent lower-body motor neuron damage [124]. Kern et al. [125] employed similar methods (FES) in spinal cord injury patients and found reverted long-term denervation-induced muscle changes via an increased muscle fiber diameter, muscle fiber area, and decreased mean percent fat. It is important to note that these results were primarily seen in type II muscle fibers, a finding which has been previously reported [126]. A potential limitation to using NMES as a countermeasure against spaceflight-induced muscle atrophy is that it appears to mainly target type II fibers [28]. However, although in a diseased population dissimilar to that of astronauts, one study [127] found increases in the proportion of type I fibers of biopsied muscles from spinal cord injury patients after a progressive 24-week general electrical stimulation regimen. Electrical stimulation also increased the muscle cross-sectional area in both fiber types. Initially, the electrical stimulation started at 15 min/day with 20 pulses, 5 s on and 5 s off, but progressively increased to 45 min/day by the study’s conclusion [127]. It should be noted these results lack external validity for the target population, astronauts, although these findings posit that electrical stimulation can provide benefits to type I muscle fibers in the appropriate context. Further, Jones [27] published a review of NMES used on adults with various advanced chronic diseases and found that most clinical trials reported that NMES improved quadricep muscle strength, muscle mass, and indices of exercise performance (i.e., six-minute walk test, endurance shuttle walk test, and cycle ergometry cardiopulmonary exercise testing). It should be reiterated that a loss of force production accompanies the loss of muscle protein, highlighting the need for NMES against spaceflight-induced muscle atrophy to improve not only muscle architecture but also function. Therefore, the conclusions of Jones [27] provide support for the idea that NMES could be a pragmatic countermeasure to LDS from a muscle functionality standpoint.

Direct microgravity or analog studies using NMES have yielded mixed results for muscle strength. However, most provide insight into its implementation. Various NMES devices have been used in orbit and in ground-based analogs, such as the Tonus-3 (Russia), Stimul-01 High Frequency (HF) set (Russia), and the Stimul-01 Low Frequency (LF) set (Russia) [135]. The information and implementation of these devices can be found elsewhere [136], though exact manufacturing details are not available. The Tonus-3 device contains separate programs designed to stimulate multiple muscle groups simultaneously at 60 Hz [28]. The Stimul-01 HF set generates high-frequency alternating electrical stimuli at 50 Hz and was designed for 40 min periods of stimulation for the lower peripheries, arms, back, shoulder, and neck muscles [137]. The Stimul-01 LF set is a wearable NMES apparatus that was placed aboard the ISS in 2006, following findings from low-frequency NMES models that elicited positive results on muscle maintenance in ground-based analogs [28,137]. This set delivers low-frequency electrical stimuli at 25 Hz. Developed by Mayr and colleagues [138], the MYOSTIM-Functional Electrical Stimulation (FES) device was also studied aboard the MIR space station and is a lower-limb wearable electromyographical NMES system that was embedded in garment fabric, capable of delivering NMES through the trousers [28,139]. Furthermore, an antagonistic hybrid training system was developed by Shiba and scientists [120] to counteract the effects of weightlessness during a 188-day ISS mission in one astronaut. It was postulated that this system was incorporated into the ISS to maintain musculoskeletal integrity by stimulating the antagonist muscle group to resist the voluntary contraction of the agonist muscle group, thereby counteracting the effects of gravity. The NMES hybrid training system was attached to the astronaut’s non-dominant arm during arm curling exercises over the last 30 days of the mission, while their dominant arm served as the control. Elbow extension power positively changed in the NMES arm (22% increase) and decreased in the control arm (−8.0%). According to magnetic resonance imaging (MRI), tricep and bicep muscle volume increased by 11.7% and 2.1%, respectively, with a concomitant increase in bone mineral density (4.6%) in the NMES arm. Lean muscle mass also increased by 10.6% in the NMES arm. Yet indices of muscle strength did not increase.

Abitante and workers [134] conducted a study for NMES spaceflight countermeasures, the purpose of which, according to the authors, was to assist in creating an NMES regimen to use during long-duration spaceflight; however, the project was conducted on competitive athletes. Stimulation was provided on the vastus medialis muscle for a total of 20 min on a 1–3 s duty cycle for a total of 300 isometric contractions. The results showed that athletes who most regularly performed endurance exercises fatigued significantly less than those who regularly performed explosive or plyometric exercises and those who did not exercise (control). Furthermore, low-frequency NMES training (STIMUL-01 LF) was tested on the quadriceps, hamstrings, tibialis anterior, gastrocnemius, and soleus muscles in gravitation unloading conditions via dry immersion [132]. Four participants completed six days of dry immersion with concurrent low-frequency (25 Hz) NMES training and with transcutaneous electrode placement, resulting in the maximal plantar flexion torque increasing by 11% and fascicle length shortening in the active NMES condition compared to a control passive condition [140]. These results were repeated in a replicate study conducted by the same study group, though two more participants were included and muscle fascicle length and pennation angle were examined [132]. The results showed that NMES could not rescue the decrease in fascicle length and alterations in the pennation angle, indications of a loss of muscle fibers [132]. Though these studies provide an idea of what occurs in muscle treated with NMES, their results lack robustness due to small sample sizes. Meanwhile, Dirks et al. [119] recruited healthy young males who were subjected to five days of knee leg-cast immobilization with or without NMES. Electrodes were placed on the quadriceps for 80 min/day, twice a day/day, at 100 Hz, with a 5 s on and 10 s off cycle. The control group experienced a 3.5% drop in the quadricep cross-sectional area (CSA) and a 9% decrease in strength. Conversely, NMES with immobilization maintained quadricep CSA, but strength dropped by 7% [119]. Gibson and colleagues [129] also implemented a leg casting model, specifically long-leg casting, in seven men who had sustained a fractured tibia and were immobilized in a cast for six weeks. Percutaneous NMES was placed on the subjects who performed 60 min/day of stimulation at a frequency of 30 Hz for 2 s on and 9 s off to elicit contraction at 5% maximal voluntary contraction. The results from the individuals with NMES were compared to those of 14 controls with similar injuries but who did not receive NMES via ultrasonography. The non-NMES quadricep CSA dropped by 17%, while those who were subjected to NMES saw a comparable CSA in the immobilized NMES leg to their uninjured control leg. No results on force output were provided. A separate leg immobilization study was conducted, in which subjects were divided into three groups. One group underwent transcutaneous NMES, while the other two groups underwent isometric exercise and a nonisometric control, respectively [130]. NMES was provided to one group on the thigh and calf muscles for 16 h/day, over 14 days, at 37 Hz, with a duration of 5 s on and 150 s off at a tolerable intensity. The NMES group saw one-half and one-fifth the amount of thigh and calf atrophy, respectively, as the other groups, which exhibited similar amounts of thigh and calf atrophy [130].

Bed rest has also been used as an analog to microgravity exposure. Multiple studies have employed these methods with NMES to evaluate its effectiveness in combating the atrophy associated with chronic or short-term bed rest. Reidy and researchers [51] studied the effects of NMES with concurrent protein ingestion on elderly, bedridden (5 days) individuals and found positive effects on muscle mass, but not on muscle strength. Duvoisin and workers [133] stimulated the dominant leg of three subjects twice a day on a three-day-on/one-day-off cycle for 30 days during bed rest, where the non-treatment leg served as a control. Electrodes were placed on the lower limbs, and NMES was administered at a total of 40 min/day (two, 20 min cycles), at 60 Hz, with a 4 s on and 16 s off cycle. The results showed that NMES delayed impairments in the torque production of the knee flexors and extensors from pre- to post-bed rest, though participants still experienced marked reductions in these markers.

Another method that has been utilized is the unloading of the limbs, as shown by Zange et al., wherein an unloading device was used to simulate microgravity exposure in conjunction with NMES intervention [131]. The study included thirteen healthy male participants who donned a Hephaistos orthosis on one leg for 60 days during all activities of daily living, with seven of them receiving NMES and protein supplementation as a countermeasure. The Hephaistos device is a specialized leg brace designed to selectively unload the calf muscles. Stimulation was applied to the soleus and lateral gastrocnemius for 40 min/day, twice/day, at 30 Hz on a 5 s on/5 s off cycle at the maximal tolerated intensity. Stimulation attenuated a net drop in the soleus and gastrocnemius muscles by 8.4% compared to the controls without NMES. Moreover, at 90-degree knee flexion, only NMES had a positive effect on torque production when compared to the controls.

It is apparent that the morphological effects of NMES on muscle architecture, while subjected to microgravity or analogs of microgravity, can be significant, while the effects on strength and indices of power or torque are mixed. In fact, it is recommended by Maffiuletti and researchers [28] to include NMES in orbit or during ground-based analog studies on the quadriceps and calf, with 60 min of NMES per day, twice/day, at a low frequency of 30 Hz, using a 5 s on/10 s off protocol. However, given the heterogeneity of stimulation modalities, intensities, duty cycles, and populations studied, the current evidence does not support prescriptive NMES protocols for spaceflight. While NMES consistently preserves muscle morphology during unloading, strength and functional outcomes remain variable.

## 5. Mechanisms of NMES That Elicit Muscle Preservation

Muscle protein balance hinges upon the relationship between MPS and MPD. Muscle is in a constant state of turnover and exhibits plasticity: in other words, the muscle’s ability to hypertrophy, degrade, or maintain in response to exogenous or endogenous factors. It is theorized that NMES can directly increase MPS [40,141]. Evidence suggests increased phosphorylation rates in mTORC1 and the subsequent activation of p70S6 protein kinase (p70S6K/S6K1) and ribosomal protein S6 after NMES administration for up to four hours [40]. When phosphorylated, these molecules drive the anabolic machinery that elicits muscle hypertrophy or, in the case of microgravity, maintains skeletal muscle. An important protein implicated in the anabolic response to NMES, due to its relationship with upstream insulin, is glycogen synthase kinase 3β (GSK3β), a constitutively active enzyme in its unphosphorylated state that naturally blunts MPS when active. When unphosphorylated, GSK3β inhibits eukaryotic initiation factor 2B (EIF2B) activity [142]. EIF2B is a protein complex that, when active, drives protein synthesis [143]. EIF2B regulates translation initiation by controlling ternary complex recycling, while eukaryotic translation initiation factor 4E (EIF4E) availability is governed by 4E-binding protein 1 (4E-BP1), which when phosphorylated releases eIF4E to promote cap-dependent mRNA recruitment. In parallel, 70 kDa ribosomal protein S6 kinase (p70S6K)—activated downstream of mTORC1—enhances translational efficiency by phosphorylating ribosomal and initiation-associated proteins, thereby synergizing with eIF2B- and eIF4E-dependent mechanisms to coordinately upregulate protein synthesis [144,145]. Akt phosphorylation directly phosphorylates GSK3β, which in turn increases EIF2B activity, thereby driving MPS as ternary complexes are synthesized at ribosomes in the cytoplasm (Figure 1) [146]. However, with microgravity exposure, insulin resistance occurs concomitantly with a rise in the ROS concentration, leading to Akt deactivation. This permits GSK3β activation, thereby delaying the formation of the ternary complex. Under normal conditions, insulin robustly activates Akt, which in turn inhibits GSK3β, thereby reducing reactive oxygen species levels, which are known to influence protein catabolism. Electrical stimulation preserves insulin sensitivity by sustaining insulin receptor substrate-1/PI3K signaling, thereby attenuating GSK3β activity. Ginjupalli and colleagues [147] supported these claims by reporting that GSK3β expression was significantly decreased in response to high-frequency electrical stimulation in rats compared to rats that did not receive the stimulation treatment.

Satellite cell stimulation from electrical stimuli has also been shown to promote myogenesis [148,149,150,151]. Satellite cells are quiescent mononucleated cells in the basolateral membrane of myocytes that, in response to certain intracellular and/or extracellular stimuli, proliferate, differentiate, and donate their nuclei to damaged or growing muscle fibers to assist in hypertrophy and myocellular regeneration (Figure 2) [149,152]. In vitro, electrical stimulation enhances the proliferation and fusion of stem cells with pre-existing myofibers by increasing the free Ca^2+^ concentration, coupled with an increased genetic expression of the myoblast determination protein 1 (MyoD), thereby promoting myoblast differentiation and growth into myotubes [149,153,154]. Myogenin, another myogenic regulation factor similar to that of MyoD, is also responsive to NMES, with previous studies showing an enhanced expression after electrical stimulation treatment [155]. These effects were also attributed to mitigating the effects that ROS have on dysregulated myofiber metabolism, which is typically seen in aging or damaged muscle. Therefore, in theory, NMES could help mitigate the effects that satellite cell recruitment has on myofibers damaged by microgravity, not only by increasing MyoD activity, but also by reducing the ROS activity typically seen in microgravity-exposed muscle. Additionally, myogenin is influenced by muscular electrical activity and induces alterations in the muscle phenotype in response to stimuli, particularly causing an increase in type II MHC after NMES [155], which adds to the growing evidence that suggests that electrical stimulation has preferential effects on type II muscle fibers. Other myogenic transcription factors such as paired box protein 3 (PAX3), paired box protein 7 (PAX7), and myogenic factor 5 (MYF5) have been shown to be unresponsive to NMES in in vitro models [151], though more research is warranted. Conversely, evidence from rats suggests that low-frequency NMES (20 Hz, 30 min, twice a day) over a 10 day period promotes satellite cell fusion and proliferation, subsequently helping to restore function and structure in muscle tissue after 10 days of treatment [148].

Additionally, researchers have explored the effects of NMES in cell culture models on focal adhesion kinases (FAKs) that interact with various focal adhesion proteins to regulate signal transduction pathways. Electrical stimulation was shown to enhance fibroblastic stress fibers, thickening them, and focal adhesions over the course of a 20 h treatment [156]. Furthermore, other signal transduction proteins, such as MAPK, c-SRC, Ras homolog family member A (RhoA), and Rho-associated protein kinase (ROCK), are upregulated when exposed to bioelectrical stimulation, promoting stress fiber formation in myocytes. Improving stress fiber formation strengthens the cytoskeleton in muscle and connective tissue, promotes cell migration and tissue remodeling, and over time enhances the force-producing capabilities of these tissues, though these need to be thoroughly assessed in in vivo models of research [156].

More recent evidence suggests a potential role for specific microRNAs (miRs) in muscle development through gene silencing via the target degradation or translational repression. Regarding microgravity, various miRs have shown downregulated expressions after microgravity exposure [72,154,157]. In short, miRs have been suggested to regulate muscle atrophy, wherein they work to limit the expression of certain proteins integral to the process [158]. miR-1 and miR-133—key regulators of muscle gene expression and myoblast proliferation—are upregulated following two weeks of NMES in a rat model of chronic obstructive pulmonary disorder (COPD), a condition characterized by severe muscle wasting and a reduced exercise tolerance [159,160]. Rats were immobilized and fitted with bilateral gastrocnemius surface electrodes (1.5 × 1.5 cm) to receive daily 30 min neuromuscular stimulation consisting of alternating 3 s trains of 100 Hz and 2 Hz pulses (0.3–1.0 ms pulse width, 2–5 mA) adjusted to elicit moderate contractions. Muscle wasting has been highlighted as an integral outcome in COPD, with afflicted individuals exhibiting lower tolerances for exercise, thus leading to muscle atrophy and bone loss in individuals who live with the disease [161]. Additionally, NMES has been shown to attenuate muscle atrophy via a modulation of the miR-486/PTEN/FoxO1 pathway, which regulates muscle protein synthesis and degradation [162]. To this end, Shen and workers [162] assessed the impact of NMES in an animal model. Electrically stimulated rats were immobilized and fitted with bilateral shaved gastrocnemius surface electrodes (1.5 × 1.5 cm) connected to a portable stimulator delivering 300 μs angular wave pulses (1 s warm-up, 2 s stimulation, 0.5 s fall time) at 100 Hz for 30 min/day, with intensity individually adjusted based on animal tolerance. The authors demonstrated that chronic hypoxia–hypercapnia rats expressed reduced levels of miR-486, and the ratio p-Akt/Akt increased, accompanied by elevated levels of TNF-α, phosphatase and tensin homolog (PTEN), Forkhead box O1 (FoxO1), atrogin-1, and MuRF1, which were reversed as a result of electrical stimulation. Overall, these findings highlight that miRs contribute an additional layer of regulation in muscle maintenance, particularly under conditions such as unloading and atrophy. In addition to their role in suppressing mRNA targets, certain miRs—including miR-1, miR-133, and the muscle-specific miR-486—also influence key signaling pathways that control both anabolic and catabolic muscle remodeling. Evidence shows that miR-1 and miR-133 affect muscle development and differentiation [163,164], while miR-486 directly targets the PTEN/FoxO1 pathway to boost PI3K/Akt signaling and reduce ubiquitin ligase levels [165,166]. Importantly, studies on NMES indicate that modulating miR-486 levels can counteract hypoxia-induced increases in PTEN, FoxO1, atrogin-1, and MuRF1, suggesting a mechanism by which miRs interact with proteolytic systems. In conjunction, functional measurements of muscular responses to NMES have to be considered. One study suggests repeated bout effects from NMES that would typically be seen in repeated bouts of other types of standard exercise modalities (i.e., resistance training). Jeon et al. [167] investigated the repeated bout effect (RBE) on muscle damage markers in untrained individuals following two bouts of low-intensity NMES to the bicep brachii. The results indicated that RBE reduced markers of muscle soreness and the pressure pain threshold after the second bout, while the maximal voluntary contraction and range of motion were unaffected. Initial increases in subjective perceptions of muscle soreness (pressure pain threshold) occurred after the first bout of NMES. Another study showed that NMES slightly elevates perceptions of delayed onset muscle soreness (DOMS) and markers of muscle damage during bed rest in young and older adults [122]. Relating to the expression of ubiquitin ligases, over prolonged periods, exercise stimulates a delayed increase in general ubiquitin conjugating activity. This increase mediates a late-phase rise in protein degradation that is required for muscle adaptation to exercise. Therefore, the ubiquitin–proteasome pathway is an essential mediator of muscle remodeling, both in atrophic states and exercise training [168]. In order to substantiate this relationship, future research should assess the influence of NMES on ubiquitin ligase expression during the potential onset of DOMS.

## 6. Future Directions

The current review highlights findings from various research foci, showcasing the potential use of NMES in astronauts during LDS. Many of the benefits discussed above are based on results from rehabilitative/injury treatment, injury prevention, performance enhancement, lower-limb disuse, and microgravity analog-based research. Future research, with an accessibility to actual microgravity settings, utilizing NMES as a countermeasure for muscle atrophy and bone resorption is necessary. Limited data from spaceflight missions are available; therefore, it is somewhat challenging to fully ascertain the positive effects of NMES on muscle and its physiology when exposed to microgravity. However, the potential for NMES to combat muscle atrophy should not be overlooked. Current NASA efforts aim to have humans reach Mars in the near future, which creates a need for mass, volume, and power constraints to minimize the costs associated with supply and fuel utilization. Additionally, the commercialization of space flight is gaining popularity with private sector companies developing their own research and development programs that focus on astronaut health and creating longevity in space. Depending on the design, NMES units are compact and require little space; however, they do require power, which is a possible logistics concern for NASA. Nevertheless, the potential benefits of NMES implementation during spaceflight need to be further explored, as little data in astronauts exist. For commercial entities, NMES can also be a possible research and treatment tool for commercial astronauts, thus generating research funds and protecting their astronauts from the deleterious effects of microgravity.

Despite its potential benefits, NMES has important limitations relative to voluntary, full range-of-motion (ROM) resistance exercise. NMES primarily induces isolated, involuntary muscle contractions and does not replicate multi-joint loading, coordinated motor control, or the neuromechanical demands achieved through full ROM exercise, all of which remain feasible during spaceflight using existing countermeasures regardless of mass and volume constraints being emphasized. Consequently, NMES should not be viewed as a replacement for resistance exercise devices such as ARED or flywheel systems. Rather, its most practical application in spaceflight may be as an adjunct modality—used to supplement traditional exercise, maintain muscle activation during periods of reduced training capacity, or mitigate atrophy when injury, fatigue, or operational constraints limit voluntary exercise. These recommendations are made in lieu of NASA’s intention to reduce mass, volume, and power needs for exercise equipment during future spaceflight missions. If future spaceflights necessitate the omission of certain currently used exercise equipment in low Earth orbit (i.e., ARED, M-MED, etc.), then implementing NMES in these missions offers a convenient adjunct countermeasure against microgravity-induced muscle atrophy. To optimize these recommendations, future research should therefore focus on defining how NMES can be optimally integrated into existing countermeasure programs, rather than evaluating it as a standalone intervention.

## 7. Conclusions

Neuromuscular electrical stimulation should be considered as a promising adjunct countermeasure to microgravity-induced losses in muscle mass. Numerous studies have demonstrated the potential benefits of NMES in reducing or delaying the onset of atrophy by attenuating muscle protein degradation. Protein turnover in the muscle is a constant process, with a balance between synthesis and degradation occurring. NMES can help shift and/or maintain the balance towards protein synthesis. The results have been mixed regarding the effect of NMES on indices of muscular strength, power, and torque, both in orbit and with ground-based analogs. Mechanisms that contribute to muscle atrophy progression should be mitigated as much as possible to maintain the astronaut’s optimal functionality during long-duration missions or EVAs. Low-frequency NMES yields the most positive benefits thus far in the current state of research. In NASA’s efforts to mitigate loads and the space taken up by exercise equipment, electrical stimulation can be applied to an astronaut performing duties integral to the shuttle or vehicle’s operation, or during full ROM exercises, due to the small size of the NMES instrument. A limitation of NMES is its preferential effects on type II muscle fibers, as astronauts typically exhibit a phenotype shift from type I to type II fibers, resulting in a net decrease in the percentage of type I fibers. An ideal situation would involve implementing an NMES unit that elicits stimuli specific to type I fibers. Lastly, more research is required to determine the effects of NMES on muscular strength and power production, as well as whether it is possible for NMES to specifically target type I fibers, especially as future missions endeavor to go further than to the moon and back.

## Figures and Tables

**Table 1:** Effects of electrical stimulation on skeletal muscle, biochemistry, and health outcomes.

Model	Intervention	ES Protocol	Study Groups	Significant Findings	Reference
**Human Diseased Models:**					
Male + female overactive bladder patients [18 individuals]	Percutaneous saphenous nerve ES	30 min weekly, 3 months; 20 Hz,	One experimental ES group	• Decreased urine urgency, nocturia, and incontinence • Improved perceptions of quality of life	[117]
Male children with MD [6 individuals]	Chronic transcutaneous LFES on TA	1 h, 3 ×/d for 7–11 weeks; 5–10 Hz	One experimental ES group Split into two phases	• Increased mean MVC of stimulated leg	[35]
Male + female MD patients [10 individuals]	Transcutaneous ES on TA	2 × 1 h/day, 2–3 months; 8 Hz or 20 Hz, 6 s on, 6 s off	One experimental group All patients received ES	• Increased in maximal torque in stimulated leg • Attenuated losses in torque in stimulated leg at 15-month follow-up	[36]
Male + female LGMD patients [24 individuals]	HVPG Transcutaneous ES on deltoid and quadriceps	5 s on, 10 s off for 10 min/d	One ES group One exercise therapy group	• Attenuated loss of deltoid muscle strength • Quadricep muscle strength similar to control group	[128]
Elderly males w/T2D [6 individuals]	Unilateral quadricep transcutaneous NMES	1 h of 500 ms pulse trains from 10 to 100 Hz	One ES leg One non-stimulated control leg	• Increased MPS • Enhanced myostatin mRNA expression	[40]
Male + female colorectal surgery patients [15 individuals]	Unilateral transcutaneous VL NMES	2 × 15 min., 30 Hz, 1 s on, 1 s off; 4 days post-op.	One ES leg One non-stimulated control leg	• Reduced loss of CSA, MT, KES • Minimal to no discomfort	[44]
Male + female post-op. CV patients [37 individuals]	Bilateral transcutaneous rectus femoris NMES	90 min 12 s on, 5 s off, POD day 3 until discharge At least 12 sessions	One NMES group One standard rehabilitation therapy group	• Increased KES compared to control at discharge • Increased right quadricep CSA	[45]
Male + female critically ill patients on MV [67 individuals]	Bilateral transcutaneous quadricep or diaphragm NMES	45 min/day until discharge: quadriceps, 50 Hz, 8 s on, 30 s off diaphragm, 30 Hz, 1 s on, 30 s off	One control group One group with ES on diaphragm One group with ES on quadriceps	• Decreased hospital stay in quadricep group • Higher respiratory muscle strength in quadricep and diaphragm groups • Improved peripheral muscle strength in quadricep group • Better functional activity scores in quadricep group	[121]
Male + female comatose patients [6 individuals]	Unilateral transcutaneous quadricep NMES	3–10 days; 2 × 30 min/day; 100 Hz, 5 s on, 10 s off	One NMES leg One non-stimulated control leg	• Maintenance of type I and II muscle fiber CSA • Increased myonuclear domain • Increased satellite cells/mm^2^	[54]
Male + female critically ill elderly ICU patients [42 individuals]	Bilateral transcutaneous proximal and distal thigh and ankle NMES	12 days of 30 min/day, 5 day/week 20 Hz, 5 s on, 2 s off	One NMES group One control group	• Reduced rate of muscle thickness loss in ICU in hospital • Reduced rate of muscle thickness loss in pre-old age group in ICU and hospital • Decrease in muscle echo intensity	[55]
**Human Injury Models:**					
Males w/fractured tibia [13 individuals]	Bilateral quadriceps percutaneous ES	1 h/day/6 weeks, 30 Hz, 2 s on, 9 s off	Two groups: One control group; control leg, immobilized leg One ES group; control leg, immobilized leg	• Increased CSA and MPS in ES legs	[129]
Male + female CTS patients [13 individuals]	Percutaneous median nerve ES	1 h 20 Hz bipolar ES post-CTRS surgery	One ES group One control group	• Increased number of innervated motor units at follow-up • Increased surface motor unit action potential • Mitigated decline in terminal motor latency in thenar muscles	[39]
Patients post-meniscectomy [10 individuals]	Unilateral upper-leg and calf transcutaneous ES	16 h/day/2 weeks; 35 Hz, 5 s on, 2.5 min off	One ES group One control isometric exercise group	• Reduced use of crutches in ES group • Attenuated loss of muscle mass and strength • Enhanced knee ROM • Reduced use of pain medication	[130]
Male athletes with ACL injury [10 individuals]	Percutaneous rectus femoris ES	5 day/week for 12 weeks of: 20 Hz, 15 s on, 10 s off; 60 min or 80 Hz, 15 s on, 75 s off; 60 min	One 20 Hz ES group One 80 Hz ES group	• 20 Hz maintained peak torque post-op. better than 80 Hz • Greater subcutaneous fat volume increase in 80 Hz	[46]
Male SCI patients [10 individuals]	2.4–9.3 years quadricep transcutaneous FES	17–25 Hz	One FES group One control group	• Increased size of myofibers • Reduced collagen, adipocyte accumulation • Facilitated myofibrillogenesis • Enhanced triad development in myofibers	[123]
Male + female SCI patients [22 individuals]	Up to 9 months quadricep transcutaneous FES	Four-phase training program (reader is directed to source)	One FES group One control group	• Increased mean fiber diameter • Increased mean percent of myofiber area • Decreased adipocyte and collagen content of muscle • Enhanced myogenesis	[125]
Male + female SCI patients [25 individuals]	2 years bilateral quadricep transcutaneous FES	Four-phase training program (reader is directed to source)	One experimental FES group	• Increase in quadriceps CSA • Increase diameter of muscle fibers • Improved force output during ES • Recovery of force allowed 25% of patients to perform ES-assisted stand-up exercises	[124]
**Human Microgravity Analog Models:**					
Male + female healthy adults [20 individuals]	5 days bed rest; bilateral quadricep NMES + protein supplementation (51 g/day)	2 × 20 min for 4 days, 75 Hz, 4 s on, 10 s off	NMES + protein group Control group	• Maintained thigh lean mass • Attenuated myostatin and MAFbx mRNA expression	[51]
Healthy young adult males [24 individuals]	5 days unilateral knee immobilization, transcutaneous quadricep NMES	5 days of 2 × 30 min/day; 100 Hz, 5 s on, 10 s off	One NMES group One control group Both groups subjected to single-leg immobilization	• No significant loss in quadriceps CSA • Decline in muscle strength • MAFbx and MuRF1 expression declined and unchanged, respectively	[119]
Male + female healthy adults [13 young, 14 older individuals]	5 days bed rest; unilateral transcutaneous quadricep NMES	5 days of 3 × 30 min/day; 60 Hz, 5 s contraction, 1 s relaxation, 10 s passive rest	One NMES leg One control leg	• Preserved voluntary muscle activation • Decrease in maximal isometric voluntary contraction • Increased CK in younger individuals	[122]
Healthy males [13 individuals]	60 days lower-limb unloading; transcutaneous soleus and gastrocnemius NMES	60 days, 2 × 20 min/day; 30 Hz, 5 s on, 5 s off	One control limb—unloaded group One NMES limb—unloaded group	• NMES mitigated triceps surae atrophy • Lower percentage decrease in knee flexion torque	[131]
Healthy males [6 individuals]	7 days DI; Transcutaneous thigh and calf NMES	5 days, 3 h/day; 25 Hz, 1 s on, 1 s off	One NMES experimental group	• Increased plantar flexion torque • Attenuated alterations in fascicle length and pennation angle of calf muscles	[132]
Healthy males [3 individuals]	30 day bedrest; unilateral transcutaneous thigh and calf NMES	3 days on, 1 day off; 2 × 20 min/day, 60 Hz, 4 s on, 16 s off	One ES leg One control leg	• Knee extensor, knee flexor, ankle flexor torque doubled in NMES leg • Attenuated losses in muscle mass, strength, and oxidative enzyme activity of NMES leg	[133]
**Human Athlete Models:**					
Male cyclists [5 individuals]	Transcutaneous VM and VL NMES	30 min, 63.3 Hz, 3.5 s on, 4.5 s off	One NMES experimental group	• Maximal “muscle pain” during NMES • Increase in serum CK 24 h post-NMES • Increase in serum lactate during NMES	[50]
Male + female athletes [33 individuals]	Transcutaneous VM NMES	20 min, 60 Hz, 1 s on, 3 s off; 300 isometric contractions	One power athlete group One endurance athlete group One control group All groups received NMES	• Endurance athletes fatigued less than explosive athletes	[134]
**Astronaut Models:**					
One male astronaut	6 months spaceflight, unilateral upper-arm transcutaneous NMES during final 4 weeks; RT w/ES using HTS	10 × 10 reps elbow curls, 3×/week; 21 V on triceps, 15.5 V on biceps	-	• Mitigated losses in elbow extension torque • Increased triceps and biceps volume • Increased BMD of upper arm • Increased muscle mass of upper arm • Reduced upper arm circumference	[120]
**Animal:**					
ICR cachexic mice	Transcutaneous ES on TA	4 days of 2 × 6 sessions/day; 100 Hz, 1 s on, 2 s off	LPS group LPS + ES group Age-matched control group	• Attenuated loss in wet weight • Attenuated increase in TNF-α • Increased CS, SDH, phospho-p38, PGC-1α expression	[34]
Swedish red cattle	Post-mortem unilateral ES on longissimus lumborum	30 min direct current 80 V stimulation	NA	• Reduced phosphorylation of CK, fructose bisphosphate aldolase, β-enolase, PK • Accelerated depletion of glycogen, PCr, ATP • Heightened meat tenderization • Faster cathepsin activity	[43]
Sprague-Dawley rats	Bilateral gastrocnemius ES post-sciatic nerve injury	30 min; 100 Hz, 5 s on, 10 s off	IR group IR + ES group Non-repaired group Control group	• Improved axon regeneration and motor functional recovery • Increased expression of LC3-II • Decreased protein p52	[47]

Bolded, underlined and highlighted cells indicate study models. ATP, adenosine triphosphate; BMD, bone mineral density; CK, creatine kinase; CS, citrate synthase; CSA, cross-sectional area; CTRS, carpal tunnel release surgery; CTS, carpal tunnel syndrome; CV, cardiovascular; DI, dry immersion; ES, electrical stimulation; FES, functional electrical stimulation; HTS, hybrid training system; HVPG, high-voltage pulse galvanic; Hz, Hertz; ICR, Institute for Cancer Research; ICU, intensive care unit; IR, immediate repair; KES, knee extensor strength; LC3-II, microtubule-associated protein 1A/1B-light chain 3-II; LF, low-frequency; LGMD, limb girdle muscular dystrophy; LPS, lipopolysaccharide; MD, muscular dystrophy; MPS, muscle protein synthesis; MT, muscle thickness; MV, mechanical ventilation; MVC, maximal voluntary contraction; NA, not applicable—unable to locate; NMES, neuromuscular electrical stimulation; PCr, phosphocreatine; PGC-1α, peroxisome proliferator-activated receptor gamma coactivator 1-alpha; phospho-p38, phosphorylated p38 mitogen activated protein kinase; PK, pyruate kinase; POD, post-operative day; ROM, range of motion; RT, resistance training; SCI, spinal cord injury; SDH, succinate dehydrogenase; T2D, type II diabetes; TA, tibialis anterior; TNF-α, tumor necrosis factor-alpha; V, Volts; VL, vastus lateralis; VM, vastus medialis.

## Data Availability

Not applicable.

## Abbreviations

The following abbreviations are used in this manuscript:

4E-BP1	4 eukaryotic initiation factor binding protein 1
Akt	Protein kinase B
ARED	Advanced Resistive Exercise Device
BFRT	Blood flow restriction training
COPD	Chronic obstructive pulmonary disorder
DOMS	Delayed onset muscle soreness
EIF2B	Eukaryotic initiation factor 2b
EIF4E	Eukaryotic translation initiation factor 4E
ERK1/2	Extracellular signal-regulated protein kinase 1 and 2
EVA	Extravehicular activities
FOXO1	Forkhead box O1
GSK-3β	Glycogen synthase kinase 3β
HDAC4	Histone deacetylase 4
HDT	Head-down tilt
HF	High frequency
IGF-1	Insulin-like growth factor 1
IRS 1/2	Insulin receptor substrate 1 and 2
ISS	International Space Station
LEO	Low Earth orbit
LDS	Long-duration spaceflight
LF	Low frequency
MAPK	Mitogen activated protein kinase
MAFbx/atrogin-1	Muscle atrophy F-box
MHC	Myosin heavy chain
MiR	miRNA
M-MED	Multi-modal Exercise Device
MPD	Muscle protein degradation
MPS	Muscle protein synthesis
mTORC	Mechanistic target of rapamycin complex 1
MuRF1	Muscle RING-finger protein 1
MYF5	Myogenic factor 5
MyoD	Myoblast determination protein-1
NASA	National Aeronautics and Space Administration
NMES	Neuromuscular electrical stimulation
p70S6K	70 kDa ribosomal protein S6 kinase
PAX3	Paired box protein 3
PAX7	Paired box protein 7
PI3K	Phosphatidylinositol 3 kinase
PTEN	Phosphatase and tensin homolog
RhoA	Ras homolog family member A
ROCK	Rho-associated protein kinase
ROS	Reactive oxygen species
SRF	Serum response factor
S6K1	Ribosomal protein S6 kinase 1
TNF-α	Tumor necrosis factor-alpha
